# Severe hemoptysis associated with lung cancer in the ICU: recurrence and outcome

**DOI:** 10.1186/s13613-025-01421-7

**Published:** 2025-03-20

**Authors:** Raphael Salvayre, Clément Hanotin, Antoine Parrot, Julien Dessajan, Alexandre Elabbadi, Nicolas Pasquier-Meunier, Muriel Fartoukh, Matthias Barral, Aude Gibelin

**Affiliations:** 1 Assistance Publique-Hôpitaux de Paris (AP-HP), Service de Médecine Intensive Réanimation, Hôpital Tenon, Sorbonne Université, 4 Rue de la Chine, 75020 Paris, France; 2 Assistance Publique-Hôpitaux de Paris, Service de Radiologie, Hôpital Tenon, Sorbonne Université, Paris, France; 3 Assistance Publique-Hôpitaux de Paris, Service de Pneumologie et Oncologie Thoracique, Hôpital Tenon, Sorbonne Université, Paris, France

**Keywords:** Hemoptysis, Bronchopulmonary cancer, Intensive care unit, Outcome

## Abstract

**Background:**

Hemoptysis is a life-threatening event in the course of lung cancer (LC). The management of the most severe episodes of hemoptysis include medical measures and vascular interventional radiology (VIR). There are few data on initial clinical and radiological features associated with early bleeding recurrence, and its prognostic significance.

**Methods:**

A monocenter retrospective study involving patients admitted to the intensive care unit (ICU) between 2009 and 2020 for severe hemoptysis (SH) associated with LC and requiring VIR.

**Results:**

During the study period, 130 patients (85% males; 59.5 ± 5.3 yrs) with SH and non-small cell (78%) or small-cell (18%) LC were analysed. SH was inaugural in half of cases. A lower respiratory tract infection (LRTI) was microbiologically documented in 39% of cases. All patients received a first-line VIR, including systemic bronchial and non-bronchial arteriography with embolisation (n = 117) and/or pulmonary arterial vaso-occlusion (n = 20). Bleeding recurred in 34% cases, after 1 day [1–3] of initial VIR attempt. Overall, the 28-day, 6-month and 12-month mortality rates were 25.3%, 47.7% and 63%, respectively. Intravenous terlipressin prior to VIR (OR 4.43, p = 0.001) and LRTI (OR 2.93; p = 0.007) were independently associated with bleeding recurrence. Tumoral cavitation (HR 3.37; p = 0.004), *Staphylococcus aureus* infection (HR 8.3; p = 0.005) and bleeding recurrence (HR 2.68; p = 0.01) were independently associated with one-year mortality.

**Conclusion:**

Lung cancer-related SH is associated with a high rate of bleeding recurrence and a poor prognosis. The association of *Staphylococcus aureus* infection with recurrence and mortality raises the potential interest of the administration of antibacterial treatment in that context.

**Supplementary Information:**

The online version contains supplementary material available at 10.1186/s13613-025-01421-7.

## Introduction

In Western countries, lung cancer (LC) has become one of the leading cause of hemoptysis [1–3]. About 20–60% patients with LC experiment episodes of hemoptysis during their clinical course, most being self-limited events. Only less than 10% of episodes are severe [4, 5], and mortality rates exceed 50% in the absence of rapid and appropriate treatment [6]. Witt et al. reported a 6-month mortality rate of 100% in LC patients with hemoptysis treated with conservative measures [7]. Significant progress has been made in the management of LC with the arrival of new treatments (immune checkpoint therapy) and in the management of hemoptysis with vascular interventional radiology. Currently, bronchial arteriography with embolisation (BAE) is the first-line therapy for severe hemoptysis (SH), allowing immediate bleeding control in most cases [8]. However, the bleeding recurrence rate could be as high as 50% [7, 8]. Recent data are available on the characteristics and management of cancer-related SH [9], but information on the predictive factors of bleeding recurrence are lacking in patients with LC after an index episode of SH.

This study was performed to identify the factors associated with bleeding recurrence in LC patients admitted to the intensive care unit (ICU) for SH, and to evaluate the mortality rates and determinants at 1 year after the index episode of hemoptysis.

## Methods

This study was conducted between April 2009 and February 2020 from a prospective database collected in a teaching referral centre for cancer and hemoptysis in Paris, France [10]. All patients admitted to the ICU for SH associated with or revealing LC and requiring vascular interventional radiology (VIR) were eligible.

**Fig. 1:**
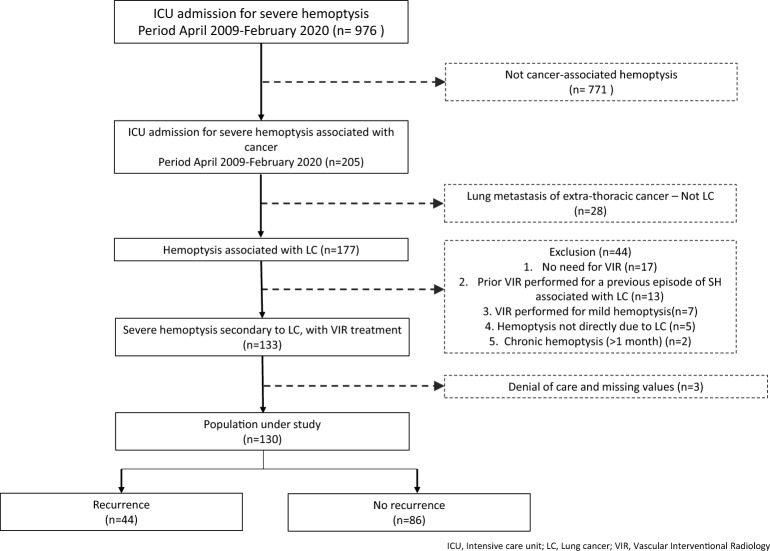
Selection of the population

Exclusion criteria were (1) No need for VIR; (2) Prior VIR performed for a previous episode of SH associated with LC; (3) VIR performed for mild hemoptysis; (4) Hemoptysis not directly due to LC; (5) Chronic hemoptysis (lasting for more than 1 month) associated with LC (Fig. 1).

### Patient characteristics and oncological status

The following demographic and clinical data were collected:(i) age, sex, alcohol consumption and smoking status, Eastern Cooperative Oncology Group—Performance Status (ECOG-PS) assessed within 1 week prior to ICU admission, comorbidities, ongoing anticoagulant or antiplatelet treatment; (ii) LC characteristics, including the histological type using the 2004 World Health Organization pathological classification [11], the LC extent using the eight TNM (tumour, node, metastasis) classification, as well as prior and current specific treatments. The cancer status was defined as controlled, in progression, recurrent or inaugural.

### Hemoptysis characteristics, management and recurrence

Hemoptysis severity was defined according to the following criteria used in our centre: (i) cumulative expectorated bleeding amount over 100 mL within 72 h; (ii) active hemoptysis with recurrence superior to 50 mL at ICU referral despite medical treatment; (iii) arterial pulmonary involvement mechanism; (iv) acute respiratory failure (clinical signs of respiratory distress, oxygen requirement over 5 L/min, or need for mechanical ventilation); (v) severe underlying chronic pulmonary disease or mandatory anticoagulation treatment. Acute illness severity was assessed using the Simplified Acute Physiology Score II (SAPS II) [12].

Hemoptysis mechanism was assessed by two senior interventional radiologists using multidetector CT-angiography (MDCTA) and VIR findings, including pulmonary angiography. Bronchial artery involvement was defined as the involvement of a dilated artery measuring more than 2 mm at the hilum [13]. Non-bronchial artery involvement was defined as abnormal dilated artery that courses into the lungs with trajectories that are not parallel to the bronchi. Pulmonary artery (PA) involvement was defined as the presence of false aneurysm, occlusion or irregularity [14]. Other CT features were collected: tumor location (central or peripheral), number of segments with alveolar consolidation, haemorrhagic bronchial impaction, and parenchymal necrosis or cavitation. Images were analysed by two senior radiologists. In case of discordance, images were reviewed by the two radiologists and a consensus was obtained.

#### Bronchial and non-bronchial systemic arteries embolisation procedure

All patients were treated in a dedicated room (GE, Innova, installed in 2012 GE 5400). When angiography showed hypertrophic arteries and/or hyperhemia of the pulmonary parenchyma, embolisation was performed with single agent or combination of tris-acryl gelatin microspheres (size 700–900 μM), coils, *N*-Butyl Cyanoacrylate Glue or Ethylene–Vinyl Alcohol Copolymer.

#### Pulmonary artery vaso-occlusion procedure

A Pigtail catheter was used to obtain a pulmonary angiography and to search for the presence of pseudoaneurysm or irregularity of the arterial walls. When indicated, vaso-occlusion was performed with stent, coils, Plug Amplatzer and/or Ethylene–Vinyl Alcohol Copolymer.

Therapeutic interventions prior to ICU admission and during ICU stay were recorded: use of intravenous terlipressin, bronchoscopic tamponade interventions, radiological vascular procedures (BAE, pulmonary arterial vaso-occlusion or stenting), and surgical lung resection.

A lower respiratory tract infection (LRTI) was defined as the combination of clinical suspicion of infection, radiological findings, and microbiological documentation (on sputum, tracheal aspirate, bronchoalveolar lavage or blood culture).

Early bleeding recurrence was defined within the first 30 days after VIR, as a new clinically significant bleeding episode after the index episode, with a volume superior to 50 mL or associated with respiratory distress [15].

### Statistical analysis

Quantitative variables were expressed as median [25th–75th percentiles], and qualitative variables as percentages. Comparisons used the Student *t* test or the Mann–Whitney *U* test for continuous variables, according to data distribution, and the number of groups according to the parametric or non-parametric distribution of the data, with Bonferroni or Dunn correction for post hoc tests. Comparisons of categorical variables used the chi-square test or Fisher exact test, as appropriate. The predictive factors of bleeding recurrence were determined by univariate and multivariable analyses using a logistic regression model. The first part of the analysis measured the crude associations (crude and adjusted odds ratio; 95% confidence intervals, (CI) between the variables of interest and the recurrence. Selection of variables for the multivariable analysis was performed by using a stepwise backward logistic regression.

Survival curves were obtained using the Kaplan–Meier method with right-censoring at the date of last follow-up information or death. Univariate and multivariate analyses were performed to identify factors associated with 1-year mortality after initial hospitalisation for SH. Variable selection method employed in the multivariable analysis was based on the approach previously described for the logistic regression model. Results were expressed as hazard ratios (HR) with 95% confidence intervals (CI) and p-values.

Statistical analyses were performed, using the Python language with the PyCharm software and R (version 4.3.2; R Core Team, Vienna, Austria, 2023).

### Regulatory and ethical issues

This study was conducted in accordance with the French regulatory requirements and was approved by the Institutional Review Board of the French learned society for respiratory medicine -Société de Pneumologie de Langue Française (CEPRO 2022-029). No specific written informed consent was required. Due to a retrospective design, we made compliance commitment with the national data recording committee (CNIL) (Compliance MR004).

## Results

### ***Patients and oncological characteristics (***Table 1***)***

**Table 1:** Baseline characteristics and outcomes of patients with Lung cancer and hemoptysis

Clinics	Missing data	All patients n = 130		Bleeding recurrence n = 44		No bleeding recurrence n = 86		*p*
Age, years	0	59.5 [55–66]		58.5 [54.8–64.5]		60 [55–66]		0.47
Gender male	0	110 (84.7)		40 (90.9)		70 (81.3)		0.16
Active smoker	2	57 (44.5)		22 (50)		35 (41.6)		0.37
ECOG-PS								
0	0	22 (16.9)		3 (8.8)		19 (22.5)		0.03
≤ 1	0	89 (68.4)		26 (59.1)		63 (73.2)		0.11
≥ 2	0	41 (31.5)		18 (40.9)		23 (26.7)		0.11
Chronic respiratory disease^a^	0	38 (29)		13 (29.5)		25 (29)		0.96
Chronic cardiovascular disease^b^	0	65 (50)		18 (40.9)		47 (54.6)		0.14
Long-term anticoagulant treatment	0	21 (16.2)		4 (9)		17 (19.7)		0.12
Long-term antiplatelet treatment	0	37 (28.5)		11 (25)		26 (30.2)		0.54
SAPS II score, points	0	25 [18.2–33.8]		28.5 [21.8–44.8]		24 [17–30]		0.05
Histology								
Non-small cell lung cancer								
- Squamous cell carcinoma	0	50 (38.4)		17 (38.6)		33 (38.4)		0.98
- Adenocarcinoma	0	40 (30.7)		16 (36.4)		24 (27.9)		0.33
- Large cell carcinoma	0	11 (8.4)		4 (9.1)		7 (8.1)		1.0
Small cell lung cancer	0	23 (17.6)		5 (11.4)		18 (20.9)		0.18
Others^c^	0	6 (4.6)		2 (4.5)		4 (4.6)		1.0
Oncological status								
Inaugural	0	67 (51.5)		24 (54.5)		43 (50)		0.63
Recurrence	0	8 (6.2)		5 (11.3)		3 (3.5)		0.08
Progression	0	29 (22.3)		8 (18.2)		21 (24.4)		0.42
Controlled	0	26 (20)		7 (15.9)		19 (22.1)		0.41
Tumor staging according TNM classification								
- T1	3	5 (3.9)		1 (2.3)		4 (4.8)		0.49
- T2	3	31 (24.4)		10 (22.7)		21 (25.3)		0.75
- T3	3	37 (29.1)		11 (25)		26 (31.3)		0.45
- T4	3	54 (42.5)		22 (50)		32 (38.8)		0.21
Metastasis	8	54 (42.5)		19 (45.2)		35 (43.7)		0.88
Hemoptysis characteristics								
Volume, mL	19^d^	200 [120–300]		250 [175–500]		155 [100–300]		< 0.01
Volume > 200 mL	19^d^	59 (53)		26 (74)		33 (43)		> 0.01
Terlipressin use prior to VIR procedure	0			15 (34)		9 (10.4)		> 0.01
Spontaneous ventilation	0	104 (80)		30 (68.2)		74 (86)		0.02
Invasive mechanical ventilation	0	25 (19.2)		13 (29.5)		12 (13.9)		0.03
LRTI associated with hemoptysis	0	49 (37.6)		24 (54)		25 (29)		> 0.01
Outcome								
Length of ICU stay, days	0	4 [2–7]		6 [3–9.2]		3.5 [2–5]		0.01
ICU mortality	0	26 (20)		14 (31.8)		12 (13.9)		0.02
6-months mortality	1	62 (47.7)		28 (73.7)		34 (39.6)		0.01
1-year mortality	1	82 (63.1)		36 (81.9)		46 (53.5)		0.01
Oncological treatment after ICU discharge	11	75 (63)		15 (37.5)		60 (75.9)		> 0.01
Time before bleeding recurrence, days	0			1 [1–3]		NC		

Continuous variables are reported as median [interquartile range (IQR) 25–75]. Categorical variables are reported as number (percentages)

*BMI* Body Mass Index, *ECOG-PS *Eastern Cooperative Oncology Group-Performance Status, *SAPS II* Simplified Acute Physiology Score, *TNM* Tumor, Node, Metastasis, *VIR* vascular interventional radiology.

^a^COPD (Chronic obstructive pulmonary disease), asthma or interstitial lung disease

^b^Heart failure, arterial hypertension, or diabetes

^c^Others include 2 sarcomatoid carcinoma, 3 lymphoid related tumor and 1 NUT midline carcinoma

^d^Hemoptysis was not quantifiable in 19 cases, with 17 patients being intubated and mechanically ventilated on admission and 2 patients being tracheotomised

During the study period, 205 patients had hemoptysis associated with LC, 130 of whom met the inclusion criteria (Fig. 1). Most were males with a median age of 59.5 years [55–66]. More than two-thirds of patients had a performance status ≤ 1. The histological cancer types were mainly non-small cell lung cancer (NSCLC) (n = 101; 78%), including squamous cell carcinoma (n = 50; 38%), adenocarcinoma (n = 40; 31%), and large cell carcinoma (n = 11; 8%). Twenty-three patients (18%) had small cell lung cancer, whereas 6 patients (5%) had another cancer type (sarcomatoid carcinoma, lymphoid tumor, NUT midline carcinoma).

Cancer was inaugural in 67 patients (52%), while it was known and either under control (n = 26; 20%), in progression (n = 29; 22%), or non-controlled (n = 8; 6%). Most patients had advanced stage of the cancer disease, with a large tumor mass classified T3 (n = 37; 28%) or T4 (n = 54; 42%) and metastasis (n = 54; 42%) (Table 1).

### Hemoptysis characteristics

The median expectorated volume of hemoptysis was 200 mL (120–300 mL) on ICU admission. Twenty-four patients (18%) had received intravenous terlipressin before ICU admission. Twenty-five patients (19%) required mechanical ventilation during the first 24 h of ICU admission. SAPS II score was 25 [IQ 18–34] (Table 1).

Initial multidetector CT angiography analysis (n = 119) showed the presence of a tumoral mass in 101 patients (85%). The mechanism of hemoptysis was related to a systemic bronchial (n = 91; 70%) and non-bronchial (n = 15; 12%) artery hypervascularisation and/or a pulmonary arterial involvement (n = 19; 15%) (Table 2).

**Table 2:** Initial imaging and vascular interventional radiology findings

	Missing data	All patients n = 130	Bleeding Recurrence n = 44	No bleeding recurrence n = 86	*p*
MDCTA	11^d^	119^*e*^	41^e^	78^e^	
Number of segments with intra alveolar consolidation	11	5 [3–9]	7 [3–10]	5 [3–8]	0.10
Endobronchial hematic impaction	11	81 (68.1)	30 (73.2)	51 (65.4)	0.39
Necrosis ou cavitation	11	35 (29.4)	17 (41.6)	18 (23)	0.04
Central mass	11	101 (84.9)	35 (85.4)	66 (84.6)	0.92
Bronchial compression	11	102 (85.7)	36 (87.8)	66 (84.6)	0.64
Arterial compression	11	85 (72.4)	32 (78.1)	53 (68)	0.25
Hypertrophy of systemic bronchial arteries^a^	11	91 (77.1)	29 (70.1)	62 (80.5)	0.23
Hypertrophy of systemic non-bronchial arteries^b^	11	15 (12.7)	8 (19.5)	7 (9.1)	0.11
Pulmonary arterial mechanism^c^	11	19 (16.1)	9 (21.9)	10 (13)	0.21
Pulmonary arterial aneurism	11	15 (12.7)	8 (19.5)	7 (9.1)	0.11
Proximal Pulmonary arterial mechanism	11	14 (11.9)	6 (15)	8 (10.4)	0.47
First VIR procedure	0	130	44	86	
Bronchial arteriography with Embolisation	0	119 (91.5)	40 (90.1)	79 (91.8)	1.0
- Duration of BAE, min	35	43.5 [30–65]	55 [38–85]	39 [28–58]	0.01
- Bronchial hypervascularisation	4^2^	106 (81.5)	36	70	0.53
- Diameter of embolised bronchial arteries (mm)	15	2.3 [1.8–3]	2.4 [1.8–3]	2.3 [1.8–3]	1.0
- Non-bronchial hypervascularisation	3	15 (10)	8 (18.1)	7 (8.1)	0.21
Pulmonary arterial vaso-occlusion	0	20 (15.4)	8 (18.1)	12 (13.9)	0.60

*MDCTA* Multidetector computed tomographic angiography, *VIR* Vascular Interventional radiology, *BAE* bronchial arteriography with embolisation

Continuous variables are reported as median [interquartile range (IQR) 25–75]. Categorical variables are reported as number (percentages)

^a^Bronchial artery involvement was defined as the involvement of a dilated artery, more than 2 mm at the hilum

^b^Non bronchial artery involvement was defined as abnormal dilated arteries that course into the lungs with trajectories that are not parallel to the bronchi

^c^Pulmonary arterial (PA) involvement was defined as the presence of false aneurysm, occlusion or irregularity of a pulmonary artery

^d^11 CT missing for lecture

^e^1 missing data due to per-interventionnal death, 3 to non-available imagery (1 only concomitant pulmonary arterial vaso-occlusion and 1 only non-bronchial hypervascularisation)

### Hemoptysis management

**Fig. 2:**
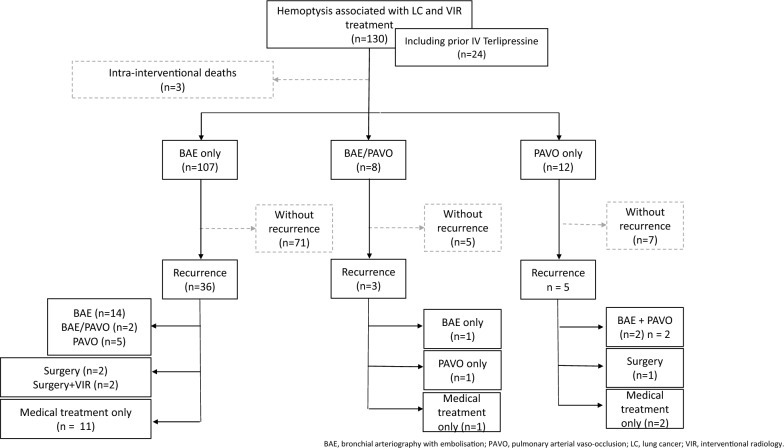
Management of severe hemoptysis associated with lung cancer

All patients were managed with a first-line attempt of VIR (Fig. 2). Altogether, 107 patients underwent a BAE of enlarged systemic arteries (median diameter 2.3 mm), 12 patients underwent a pulmonary arterial vaso-occlusion, and 8 patients had both. First-line VIR procedure was not completed three times, due to episodes of fatal hemoptysis of pulmonary arterial mechanism. Additional procedures of pulmonary arterial vaso-occlusion were required (pulmonary arterial mechanism not identified during the first procedure, and finally diagnosed at recurrence in 7 patients). Finally, pulmonary arterial vasculature was involved in 30 patients.

### Hemoptysis and infection

**Fig. 3:**
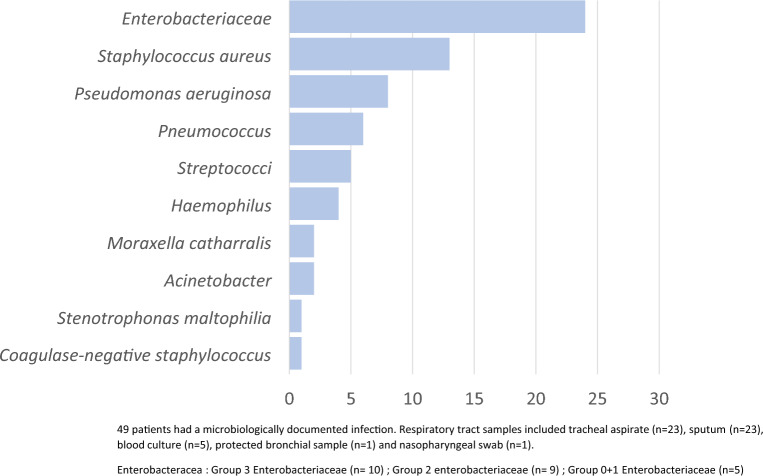
Microbiological documentation of episodes of severe hemoptysis associated with lung cancer

A lower respiratory tract infection was clinically suspected in 96 patients. A microbiological documentation was obtained in 49 of them: bacteria (n = 47), mycobacteria (*Mycobacterium intracellulare,* n = 1), virus (coronavirus with *Pseudomonas aeruginosa* coinfection, n = 1) and fungi (Aspergillus fumigatus, n = 1). *Staphylococcus aureus* (n = 13) and group 3 *Enterobacterales* (n = 10) were the most frequent bacterial species (Fig. 3). Pulmonary arterial involvement was frequently associated with infection (n = 18/30; 60%).

### Bleeding recurrence and mortality

Bleeding recurred early in 44 patients (33.8%), after 1 day [1–3] of initial attempt of VIR. Median bleeding recurrence volume was 100 mL [40–1000]. Recurrence was associated with clinical worsening: acute respiratory failure (n = 9; 20%), need for mechanical ventilation (n = 7; 16%), and cardiac arrest (n = 11; 25%). As compared with their counterparts, the patients with bleeding recurrence had a greater use of intravenous terlipressin before VIR (n = 15; 34% vs. n = 9; 10%; p = 0.001), a higher SAPS II score (29 [22–45] vs. 24 [8–30]; p = 0.05), a higher initial bleeding amount (250 mL [175–500] vs. 155 mL [100–300]; p = 0.03), and higher rates of non-bronchial systemic arteries involvement (n = 8; 18% vs. n = 7; 8%; p = 0.21), pulmonary arterial vaso-occlusion (n = 8; 18% vs. n = 12; 14% p = 0.60), parenchymal cavitation (n = 17; 42% vs. n = 18;23% p = 0.04) and need for initial mechanical ventilation on admission (n = 13; 30% vs. n = 12; 14%; p = 0.03) (Tables 1, 2).

In univariate analysis, bleeding recurrence was associated with performance status, initial bleeding amount, intravenous administration of terlipressin before VIR, duration of BAE procedure, pulmonary arterial mechanism, and LRTI on ICU admission. In multivariable analysis, bleeding recurrence was independently associated with a good performance status (OR = 0.2; 95% CI, 0.04–0.79; p = 0.02), the intravenous administration of terlipressin before VIR (OR = 4.71; 95% CI, 1.74–12.71; p = 0.002), and a microbiologically documented LRTI (OR = 3.55; 95% CI, 1.55–8.1; p = 0.002) (Table 3).

**Table 3:** Univariate and multivariable analyses of predictive factors of early bleeding recurrence

	Total n	Bleeding recurrence n	Univariate analysis OR [CI 95%]		p	Multivariable analysis OR [CI 95%]		p
Performance status 0								
No	108	41	1					
Yes	22	3	0.26 [0.07–0.92]	0.03		**0.198 [0.04–0.79]**		**0.02**
Hemoptysis volume > 200 ml								
No	56	9	1					
Yes	55	26	2.32 [1.55–9.10]	0.03		–		–
IMV on ICU admission								
No	105	31	1					
Yes	25	13	2.59 [1.06–6.29]	0.06		–		–
Terlipressin before VIR procedure								
No	106	29	1					
Yes	24	15	4.43 [1.74–11.21]	> 0.001		**4.71 [1.74–12.71]**		**0.002**
Necrosis ou cavitation								
No	84	24	1					
Yes	35	17	2.36 [1.04–5.33]	0.06		–		–
Diameter of embolised bronchial arteries > 2.3 mm^a^								
No	52	12	1					
Yes	51	23	2.03 [0.96–4.23]	0.06		–		–
Duration of BAE > 43.5 min^b^								
No	42	11	1					
Yes	42	20	2.42 [1.12–5.21]	0.03		–		–
Pulmonary arterial mechanism^c^								
No	100	29	1					
Yes	30	15	2.45 [1.06–5.64]	0.05		–		–
LRTI on ICU admission								
No	81	20	1					
Yes	49	24	2.93 [1.37–6.22]	0.01		**3.55 [1.55–8.1]**		**0.003**

*IMV* invasive mechanical ventilation, *ICU* intensive care unit; VIR, Vascular Interventional radiology; LRTI, Lower respiratory tract infection

^a^Median diameter of embolised bronchial arteries during first BAE attempt is 2.3 mm

^b^Median duration of the first BAE procedure is 43.5 min

^c^Pulmonary arterial mechanism: 20 identified during the first VIR procedure, 7 during the second procedure and 3 died before the end of first VIR procedure

**Fig. 4:**
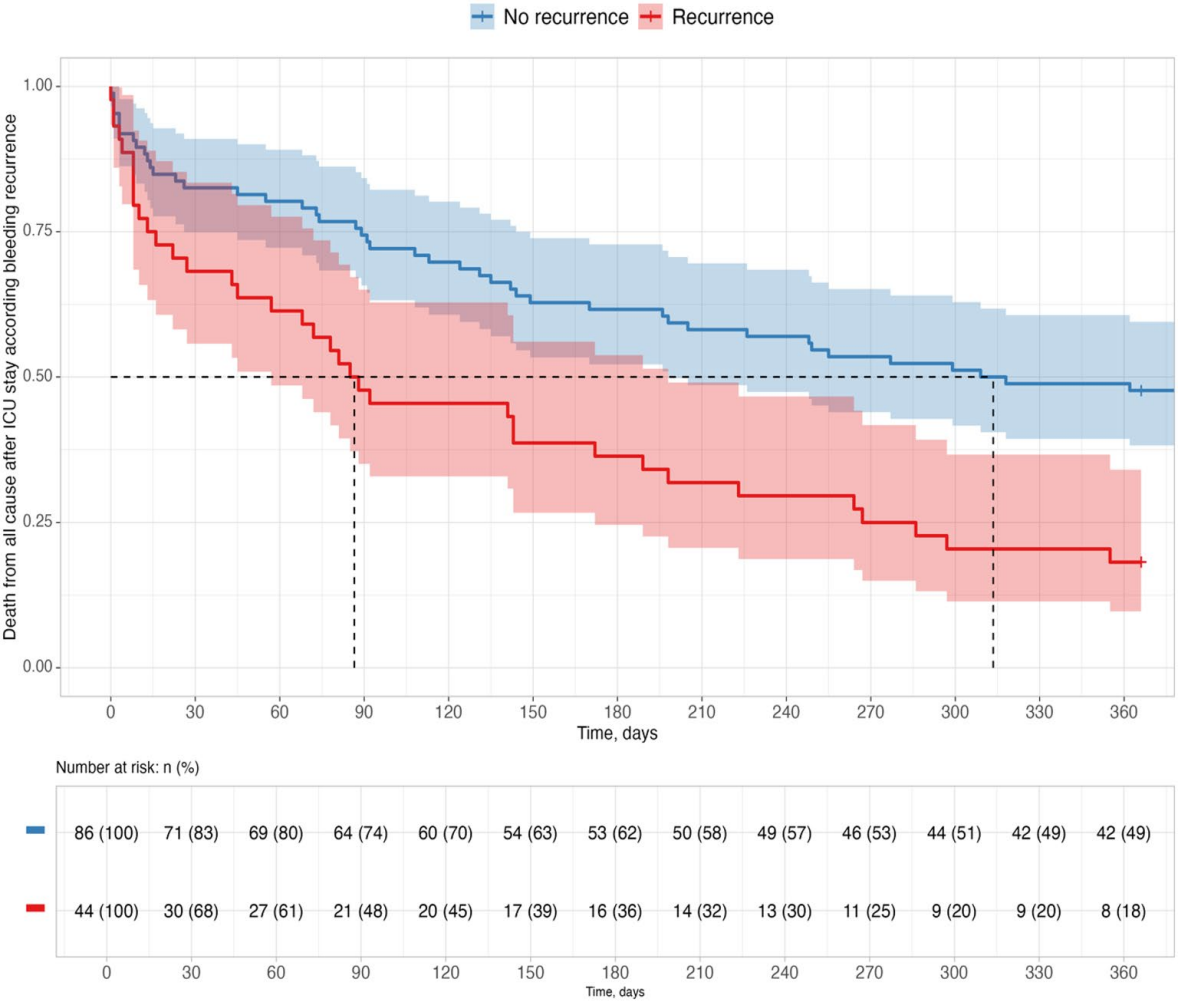
Kaplan Meier graph of the probability of one-year survival from ICU admission in patients with (red curve) and without (blue curve) bleeding recurrence

Overall, the 28-day, 6-month and 12-month mortality rates were 25.3%, 47.7% and 63%, respectively (Fig. 4). Those rates were higher in case of bleeding recurrence, respectively 38.6%, 63.6% and 81% (Table 1). The variables associated with 1-year mortality are detailed in Table 4. In multivariable analysis, CT parenchymal necrosis or cavitation (HR = 1.82; 95% CI, 1.03–3.22; p = 0.0043), *Staphylococcus aureus* infection (HR = 3.20; 95% CI, 1.57–6.53; p = 0.003), and bleeding recurrence (OR 2.04 [IC 1.17–3.54]; p = 0.012) were associated with 1-year mortality. Inaugural hemoptysis as a primary manifestation of lung cancer was associated with a higher survival rate at 1 year in univariate analysis (HR = 0.50; 95% CI, 0.32–0.78; p = 0.002).

**Table 4:** One year mortality of 130 patients with severe hemoptysis associated with lung cancer: univariate and multi variable analyses

	Total n	1-year mortality n		Univariate analysis HR [95% CI]	p	Multivariable analysis HR^a^ [95% CI]	P
Performance status							
PS ≤ 1	89		33	1			
PS ≥ 2	41		29	2.71 [1.84–9.11]	< 0.001	–	–
Inaugural hemoptysis							
No	63		39	1			
Yes	67		23	0.50 [0.32–0.78]	0.002	**0.83 [0.42–1.63]**	**0.6**
Tumoral mass T4							
No	73		29	1			
Yes	54		33	1.73 [1.11–2.69]	0.015	–	–
Cancer progression							
No	101		78	1			
Yes	29		22	2.73 [1.70–4.39]	< 0.001	**2.13 [0.98–4.63]**	**0.057**
IMV at ICU admission							
No	105		43	1			
Yes	25		19	2.47 [1.48–4.10]	0.001	–	–
Central tumoral mass							
No	18		15	1			
Yes	101		85	2.51 [1.09–5.79]	0.031	–	–
Necrosis or cavitation							
No	84		33	1			
Yes	35		24	2.65 [1.65–4.26]	< 0.001	**1.82 [1.03–3.22]**	**0.043**
Pulmonary arterial mechanism							
No	100		41	1			
Yes	30		21	2.59 [1.62–4.15]	< 0.001	–	–
SA-documented LRTI							
No	83		33	1			
Yes	13		11	4.34 [2.24–8.40]	< 0.001	**3.20 [1.57–6.53]**	**0.003**
Early bleeding recurrence (< 1 month)							
No	86		34	1			
Yes	44		28	2.22 [1.43–3.45]	< 0.001	**2.04 [1.17–3.54]**	**0.012**

*PS* performance status, *VIR* *IMV* invasive mechanical ventilation, *LRTI* lower respiratory tract infection, *SA* *Staphylococcus aureus*, *HR* hazard ratio, *CI* confidence interval

^a^Median diameter of embolised bronchial arteries during first BAE attempt is 2.3 mm

After ICU discharge, oncological treatments were administered to 75 patients (57%), including chemotherapy (n = 52), immunotherapy (n = 13), radiotherapy (n = 12), and surgery (n = 12). Information was missing in 11 patients. Noteworthy, 60 patients (46%) without bleeding recurrence received specific treatment, as compared with 15 patients (11%) with recurrence (p < 0.001).

## Discussion

In this study, we aimed at assessing the rate of bleeding recurrence in lung cancer patients admitted to the ICU for SH and treated with first-line vascular interventional radiology, identifying the factors associated with bleeding recurrence, and assessing its prognostic signification. The main findings were the following: bleeding recurred in 34% cases, after 1 day [1–3] of initial attempt of VIR. The 1-yearmortality was 63.1% and over 81.9.6% in case of bleeding recurrence. A good performance status, the administration of intravenous terlipressin prior to VIR and respiratory infection on admission were independently associated with bleeding recurrence. Tumoral cavitation, *Staphylococcus aureus* infection and bleeding recurrence were independently associated with 1-year mortality.

Bleeding recurrence after an index episode of hemoptysis and its determinants are poorly investigated, with reported rates ranging from 18 to 40% [14, 16, 17]. This variability may be explained by small samples of patients with LC, and by different initial bleeding severity and management reported in the literature [14, 16, 17]. Furthermore, the date of bleeding recurrence and its amount are often poorly or not defined. As suggested by Fartoukh et al. [15], we defined the time of bleeding recurrence at 1 month after the index episode to avoid recurrence related to mechanisms of revascularisation via tumoral angiogenesis [18, 19]. In our study, 34% of patients had bleeding recurrence within one month after a first-line VIR attempt, and bleeding recurred very early most of the time, as already described [16]. Several factors associated with bleeding recurrence have been identified, including etiology and mechanism of hemoptysis [16, 20–23]. These observations are indicative of the VIR procedure complexity. Our findings indicated that the non-bronchial systemic arterial vasculature and the pulmonary arterial vasculature were frequently involved, and may have contributed to the bleeding recurrence The intravenous administration of terlipressin before VIR was identified as an independent risk factor of bleeding recurrence. Despite its huge utility for the management of massive hemoptysis [24], terlipressin may generate per-procedural technical difficulties related to vasospasm interfering with the arteries visualisation, catheterism or embolisation, thus leading to an incomplete or failed VIR procedure [20]. Few data are available on this issue, except during post-partum haemorrhage and uterine embolisation [25].

Bleeding recurrence was associated with clinical worsening. It was also associated with high mortality rates at 28 days, 6 months and one year, close to those of patients with LC hospitalised in the ICU, all causes combined [26, 27]. However, it should be noted that two thirds of patients surviving the ICU stay were able to receive oncological treatments, especially those who had no bleeding recurrence. Altogether, these findings support the large policy of ICU admission of lung cancer patients with SH, with early reevaluation of life sustaining therapies, especially in patients with bleeding recurrence. Tumoral necrosis or cavitation and a poor performance status were independently associated with 1-year mortality, as reported by others [9, 10]*,* while inaugural bleeding was protective. At 1 year, the mortality rate was slightly lower than that reported by Razazi et al. [10], probably related to the new targeted therapies and immunotherapy that were developed in the meantime, and from which some patients may quickly benefit [28].

A good performance status (PS 0) was a protective factor of recurrence. This observation might be linked to the predominance of inaugural cancers in this subgroup characterised by the absence of vascular remodelling linked to radiotherapy, chemotherapy or a previous infection. Likewise, inaugural LC was a protective factor of mortality in univariate analysis. After adjustment for cancer progression at 1 year, this variable did not remain protective, suggesting that the diagnosis of LC during hemoptysis is more closely related to the fact of having a cancer that is naïve to oncologic treatment.

Our findings suggest the role of LRTI as a risk factor for bleeding recurrence and mortality. In our study, the microbiological spectrum of bacterial species was similar to that reported by Carteaux et al. [29], with the identification of *Staphylococcus aureus*, *Pseudomonas aeruginosa*, and *Streptococcus pneumoniae.* In addition, our study pointed out the presence of *group 3 Enterobacterales* (20% vs 6.5%), while no anaerobic bacteria were detected. The links between respiratory infection and LC are poorly described [30–32]. Interestingly Khono et al. [32] described the pattern of respiratory infection in patients with LC, highlighting the presence of *Klebsiella pneumoniae* which was comparable to our work. In our study, lower respiratory tract infection was an independent risk factor of bleeding recurrence, and *S. aureus* an independent risk factor of mortality. Among the elements that can explain this severity, we highlight a possible link between arterial pulmonary mechanism and LRTI.

### Limits of the study

Several limitations may be discussed. First, the retrospective nature of our study is associated with the fact that several data are missing; second, the skill of our center in the fields of thoracic oncology and hemoptysis may lead to a bias recruitment of patients with more complex or rare situation; third, the prognosis of patients may have changed throughout time, as new innovative oncological treatments have been developed during the study period.

In conclusion, SH associated with LC is associated with a high rate of early bleeding recurrence despite a first-line attempt of VIR, and a poor prognosis at 6 months and one year. Respiratory tract infection, particularly *S. aureus* infection, is associated with bleeding recurrence and mortality. The place of initial antimicrobial treatment should be further investigated in episodes of SH associated with LC.

## Supplementary Information

Supplementary Material 1.

Supplementary Material 2.

## Data Availability

The datasets used and/or analysed during the current study are available from the corresponding author on reasonable request.

## Abbreviations

BAE: Bronchial arteriography with embolization
CEPRO: Comité d’Evaluation des Protocoles de Recherche Observationnelle
CI: Confidence interval
CNIL: Commission Nationale de l'Informatique et des Libertés
CT: Computed tomography
ECOG-PS: Eastern Cooperative Oncology Group-Performance Status
GE: General electric
HR: Hazard ratios
ICU: Intensive care unit
IV: Intraveinous
LC: Lung cancer
LRTI: Lower respiratory tract infection
MDCTA: Multidetector computed tomography angiography
MR004: Méthodologie de Référence 004
OR: Odds ratio
PA: Pulmonary involvement
PAVO: Pulmonary arterial vaso-occlusion
SA: *Staphyloccus aureus*
SAPS II: Simplified Acute Physiology Score II
SH: Severe hemoptysis
TNM classification: Tumor node metastasis classification
VIR: Vascular interventionnal radiology
