# Supplementary material for: Severe hemoptysis associated with lung cancer in the ICU: recurrence and outcome
Source: Ann Intensive Care. 2025 Mar 20;15:33. doi: 10.1186/s13613-025-01421-7 (PMC11925840; doi:10.1186/s13613-025-01421-7)
Supplement: Supplementary file 1 — Supplementary Material 1. [file 13613_2025_1421_MOESM1_ESM.pptx]

## Slide 1
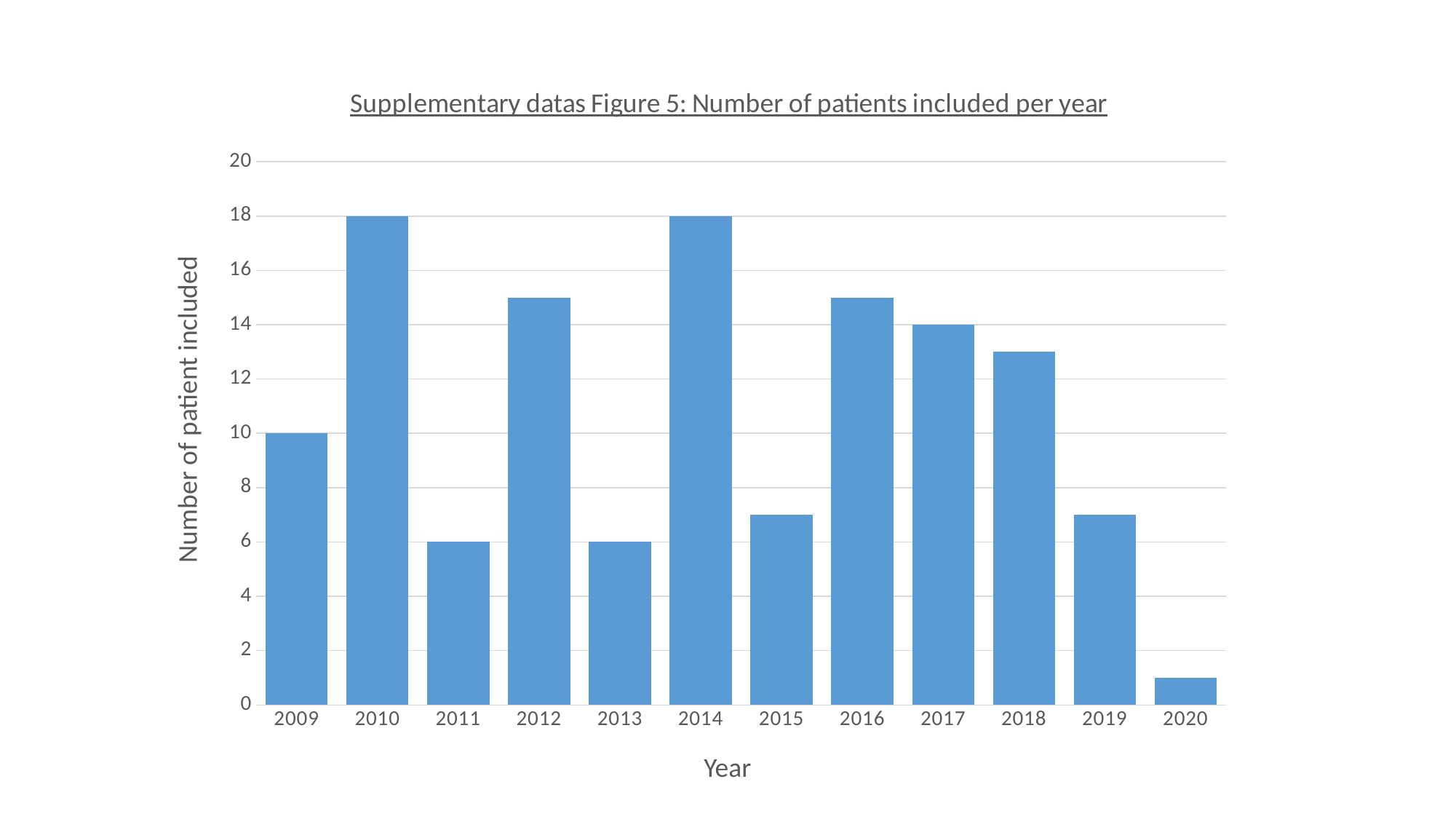

### Chart: Supplementary datas Figure 5: Number of patients included per year
| Category | Number of patient included |
|---|---|
| 2009 | 10.0 |
| 2010 | 18.0 |
| 2011 | 6.0 |
| 2012 | 15.0 |
| 2013 | 6.0 |
| 2014 | 18.0 |
| 2015 | 7.0 |
| 2016 | 15.0 |
| 2017 | 14.0 |
| 2018 | 13.0 |
| 2019 | 7.0 |
| 2020 | 1.0 |Number of patient included
Year
