# Supplementary material for: Severe hemoptysis associated with lung cancer in the ICU: recurrence and outcome
Source: Ann Intensive Care. 2025 Mar 20;15:33. doi: 10.1186/s13613-025-01421-7 (PMC11925840; doi:10.1186/s13613-025-01421-7)
Supplement: Supplementary file 2 — Supplementary Material 2. [file 13613_2025_1421_MOESM2_ESM.docx]

**Supplementary data.**

**Severe Hemoptysis associated with Lung Cancer in the ICU: recurrence and outcome.**

Raphael Salvayre^1^, Clément Hanotin^2^, Antoine Parrot^3^, Julien Dessajan^1^, Alexandre Elabbadi^1^, Nicolas Pasquier-Meunier^1^, Muriel Fartoukh^1^, Matthias Barral^2^, and Aude Gibelin^1^

**Authors Affiliation:**

^(1)^ Sorbonne Université, Assistance Publique – Hôpitaux de Paris (AP-HP), Service de médecine intensive réanimation, Hôpital Tenon, Paris, France

^(2)^ Sorbonne Université, Assistance Publique – Hôpitaux de Paris, Service de radiologie, Hôpital Tenon, Paris, France

^(3)^ Sorbonne Université, Assistance Publique – Hôpitaux de Paris, Service de pneumologie et oncologie thoracique, Hôpital Tenon, Paris, France

**Supplementary Table 1: Multivariate analysis of predictive factors of early bleeding recurrence**

|  | **HR**^1^ | **95% CI**^1^ | **p-value** |
| --- | --- | --- | --- |
| Performance Status 0 | 0.31 | 0.10, 1.01 | 0.021 |
| LRTI on ICU admission | 2.27 | 1.25, 4.11 | 0.008 |
| Terlipressin before IR procedure | 2.86 | 1.53, 5.36 | 0.002 |
| ^1^HR = Hazard Ratio, CI = Confidence Interval | | | |

**Supplementary Table 2: Respiratory microbiological sampling and documentation according to 1-year mortality**

|  | No N = 68*^1^* | Yes N = 62*^1^* |
| --- | --- | --- |
| No lower respiratory tract sample at admission | 16 (25%) | 18 (29%) |
| LRTI on ICU admission | 13 (27%) | 31 (38%) |
| SA-documented infection | 0 (0%) | 13 (29%) |
| *^1^* n (%) |  |  |
